# Taxonomic Identity of *Carpinus dayongina* Franchet (Betulaceae)

**DOI:** 10.3897/phytokeys.177.57725

**Published:** 2021-05-12

**Authors:** Qianqian He, Runan Zhao, Anguo He, Zunling Zhu, Yihua Tong

**Affiliations:** 1 College of Landscape Architecture, Nanjing Forestry University, Nanjing 210037, China Nanjing Forestry University Nanjing China; 2 Zhejiang Dapanshan National Natural Reserve, Pan’an 322300, China South China Botanical Garden, Chinese Academy of Sciences Guangzhou China; 3 College of Art & Design, Nanjing Forestry University, Nanjing 210037, China Nanjing Forestry University Nanjing China; 4 Key Laboratory of Plant Resources Conservation & Sustainable Utilization/Key Laboratory of Digital Botanical Garden of Guangdong Province, South China Botanical Garden, Chinese Academy of Sciences, Guangzhou 510650, China South China Botanical Garden, Chinese Academy of Sciences Guangzhou China

**Keywords:** Hornbeam, new synonym, taxonomy

## Abstract

*Carpinus polyneura* and *C. dayongina* are recognised as separate species in *Flora of China*. In this study, the results of an examination of literature, morphological comparison and phenetic clustering of nuclear ITS sequences suggest that *C. dayongina* is conspecific with *C. polyneura*. Thus, we propose reducing *C. dayongina* to a synonym of *C. polyneura*.

## Introduction

*Carpinus* (Linnaeus 1753) is a large genus in the family Betulaceae. It contains about 50 species distributed across the Northern Hemisphere (Asia, Europe, North America) (Li and Skvortsov 1999; Holstein and Weigend 2017), 36 of which occur in China (including 30 endemic species) (Li and Skvortsov 1999; Tong et al. 2014; Lu et al. 2017; Lu et al. 2018; Lu 2020). Although some *Carpinus* species have been studied (Hu 1948; Hu 1964; Li 1979; Ill and Chang 1997; Li and Skvortsov 1999), this genus is still taxonomically problematic because of the lack of comprehensive field investigations and analyses of morphological characters for some species.

**Figure 1.**
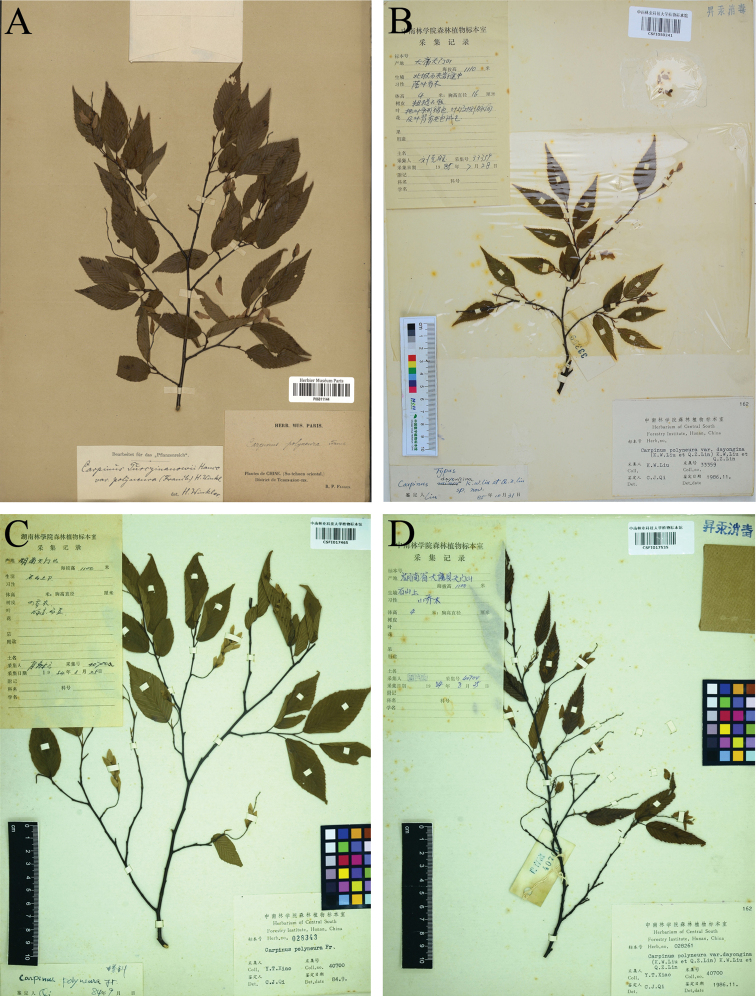
Specimens of *Carpinus polyneura* Franchet **A** lectotype of *C. polyneura* (*P. G. Farges s.n.*, P06811144) **B** holotype of *C. dayongina* K. W. Liu & Q. Z. Lin (*K. W. Liu 33359*, CSFI050241) **C** paratype of *C. dayongina* (*Y. T. Xiao 40700*, CSFI017465) **D** paratype of *C. dayongina* (*Y. T. Xiao 40700*, CSFI017535).

*Carpinus polyneura* Franchet was described, based on collections (*Farges*, *s.n.*) (Fig. 1A) from Chengkou County, Sichuan Province (now Chengkou County, Chongqing City) (Franchet 1899). Although there are some morphological variations amongst populations of this species due to its wide distribution, the higher density of leaf veins and setiform serrate leaf margin make it clearly distinguishable from the other species of *Carpinus* (Hu 1964).

In the protologue, *Carpinus polyneura* is described as having lanceolate or ovate-lanceolate with a long-acuminate apex and simply serrate margin and nutlets that are mainly villous at the apex. *Carpinus dayongina* K. W. Liu & Q. Z. Lin was described, based on several collections (Fig. 1B–D) from Tianmenshan, Dayong County, Hunan Province (now Tianmenshan, Zhangjiajie City, Hunan Province) (Liu and Lin 1986). Liu and Lin (1986) stated that *C. dayongina* was similar to *C. polyneura*, but could be distinguished by its narrower leaves, shorter infructescence, smaller bracts and narrower leaves. They cited two collections, i.e. *K. W. Liu 33359* and *Y. T. Xiao 40700*, with the former designated as holotype. The collection, *Y. T. Xiao 40700*, contains a total of six specimens (here considered as duplicates). The specimen with the barcode CSFI017465 (Fig. 1C) bears leaves that are clearly wider in shape (ovate-lanceolate) than those of the other five specimens (narrow-lanceolate) and it was identified as *C. polyneura* previously (by Qi Cheng Jing in June 1984).

Li and Skvortsov (1999) pointed out that *C. polyneura* has leaves with doubly setiform serrate margin and nutlets that are pubescent, while *C. dayongina* has narrower leaf blades with simply setiform serrate margin and nutlets that are only villous at the apex (Table 1).

**Table 1.** Differences between *Carpinus polyneura* and *C. dayongina* indicated by Li and Skvortsov (1999).

Species	Leaf	Length × width (cm)	Margin of leaf	Nutlet
*C. polyneura*	Elliptic-lanceolate or oblong-lanceolate	4–8 × 1.5–2.5	Doubly setiform serrate	Pubescent
*C. dayongina*	Lanceolate or ovate-lanceolate	2.5–4.5 × 1–1.5	Simply setiform serrate	Only villous at apex

When revising the species of *Carpinus* in China, we noticed that these two species are very similar to each other. This made us speculate that the two are possibly conspecific, although Li and Skvortsov (1999) followed Liu and Lin (1986) and treated them as separate species in *Flora of China*.

## Materials and methods

### Morphological analysis

Specimens of *C. polyneura* and *C. dayongina* deposited in the herbaria CSFI, HHBG, HIB, HNWP, IBK, IBSC, IFP, KUN, LBG, NAS, P, PE, SHM, SZ and WUK were studied and field investigations in Guizhou, Hubei, Hunan and Zhejiang to study *C. polyneura* and *C. dayongina* had been conducted in recent years. The morphological characteristics of the two species were also documented via photography and some of the physical features were measured (Table 2). Abbreviations for the names of herbaria in this study refer to the Herbarium Index Database (http://sweetgum.nybg.org/science/ih/).

**Table 2.** Specimens used for measurement of morphological characters of *Carpinus polyneura* and *C. dayongina*.

Species	Collector	Collection number	Collection site	Herbarium
*C. polyneura*	T. L. Dai	104469	Chengkou, Chongqing	PE
	H. F. Zhou	26421	Fengjie, Chongqing	KUN
	H. F. Zhou	26317	Fengjie, Chongqing	KUN
	Z. R. Zhang	25054	Fengjie, Chongqing	KUN
	Y. Liu	668	Shennongjia, Hubei	KUN
	P. C. Cai	20297	Shimen, Hunan	CSFI
	C. L. Long	87290	Shimen, Hunan	CSFI
	J. R. Zheng	80108	Shimen, Hunan	CSFI
	P. C. Cai	20442	Zhangjiajie, Hunan	CSFI
	P. Y. Li	8277	Langao, Shaanxi	KUN
	T. P. Soong	39145	Baoxing, Sichuan	KUN
	G. H. Yang	57149	Emeishan, Sichuan	KUN
	W. P. Fang	7546	Emeishan, Sichuan	PE
	W. P. Fang et al.	31110	Emeishan, Sichuan	IBK
	W. P. Fang et al.	32888	Emeishan, Sichuan	IBSC
	G. H. Yang	54569	Emeishan, Sichuan	IBSC
	S. G. Wu	394	Sichuan	KUN
	G. R. Chen	2383	Tiantai, Zhejiang	KUN
	Anonymous	2759	Without precise locality	KUN
	S. Y. Hu	1906	Without precise locality	KUN
*C. dayongina*	Y. T. Xiao	40700	Tianmenshan, Hunan	CSFI
	K.W. Liu	33359	Tianmenshan, Hunan	CSFI, PE
	Q. P. Zhang	2020072801	Tianmenshan, Hunan	NF
	Q. P. Zhang	2020072802	Tianmenshan, Hunan	NF

### Molecular analysis based on nuclear ribosomal ITS sequences

Twelve individuals from five populations of the two species (Table 3), including nine individuals of *C. polyneura* (P1–P9) and three individuals from the type locality of *C. dayongina* (D1–D3), respectively, were sampled. Fresh leaves were collected from each individual. Coordinates and altitude information were recorded by using a hand-held GPS. All voucher specimens were stored in Nanjing Forestry University (NF).

**Table 3.** Geographical information of four populations of *Carpinus polyneura* and one population of *C. dayongina* used for phylogenetic analyses of ITS sequences.

Species	Number of individuals	Collection site	Latitude (N) / Longitude (E)	Altitude (m)
*C. polyneura*	4 (P1–P4)	Hupingshan, Hunan	30°0'42.1"N, 110°36'20.5"E	350
	3 (P5–P7)	Dapanshan, Zhejiang	29°12'33.4"N, 120°43'56.6"E	600
	1 (P8)	Liujiaping, Hubei	30°6'27.9"N, 110°43'56.6"E	1380
	1 (P9)	Caoyuan, Guizhou	26°18'43.9"N, 106°54'39.6"E	1460
*C. dayongina*	3 (D1–D3)	Tianmenshan, Hunan	29°3'5.8"N, 110°28'30.3"E	1335

DNA was extracted using the modified CTAB method (Doyle and Doyle 1990). PCR amplifying and sequencing of the ITS fragment refer to Lu et al. (2016). We made an alignment of 12 newly-sequenced ITS fragments and 10 ITS sequences of *C. polyneura*, *C. mollicoma* (Hu 1949), *C. rupestris* (Camus 1929) and *C. tschonoskii* (Maximowicz 1881b) that were downloaded from NCBI (Table 4). We used Mega X (Kumar et al. 2018) to construct a neighbor-joining (NJ) tree (Saitou and Nei 1987) using pairwise deletion and the *P*-distance model. Bootstrap values were set to 1000 to calculate the support values.

**Table 4.** ITS sequences used in this study are from NCBI; a dash (–) indicates missing data.

Species	Location	Voucher	ITS from NCBI
*C. polyneura* (P1)	Hupingshan, Hunan	Z.F. Chen 20200601	MW882972
*C. polyneura* (P2)	Hupingshan, Hunan	Z.F. Chen 20200603	MW882973
*C. polyneura* (P3)	Hupingshan, Hunan	Z.F Chen 20200605	MW882974
*C. polyneura* (P4)	Hupingshan, Hunan	Z.F. Chen 20200607	MW882975
*C. polyneura* (P5)	Dapanshan, Zhejiang	A.G. He 20200710	MW882976
*C. polyneura* (P6)	Dapanshan, Zhejiang	A.G. He 20200715	MW882977
*C. polyneura* (P7)	Dapanshan, Zhejiang	A.G. He 20200717	MW882978
*C. polyneura* (P8)	Liujiaping, Hubei	Z.Q. Lu LJP-1	MW882979
*C. polyneura* (P9)	Caoyuan, Guizhou	Z.Q. Lu CY-1	MW882980
*C. dayongina* (D1)	Tianmenshan, Hunan	Q.P. Zhang 2020072801	MW893478
*C. dayongina* (D2)	Tianmenshan, Hunan	Q.P. Zhang 2020072802	MW893479
*C. dayongina* (D3)	Tianmenshan, Hunan	Q. P. Zhang 2020072803	MW893480
*C. polyneura*	Xingshan, Hubei	Chen et al. 961325	AF081517
	–	Liu 631	FJ011726
	Tiantai Mt., Zhejiang	–	JF796533
	Qinling Mt.	SZH 454	MH703152
	Qinling Mt.	Q875	MH711693
*C. mollicoma*	Daba Mt. Shaanxi	–	KX946977
*C. rupestris*	Daba Mt. Shaanxi	–	KX946978
*C. tschonoskii*	Hangzhou Bot. Gard., Zhejiang	W 97-30	AY006369
	–	Lee s. n.	FJ011733
	Qinling Mt.	HZ 283	MH710986

## Results and discussion

**Figure 2.**
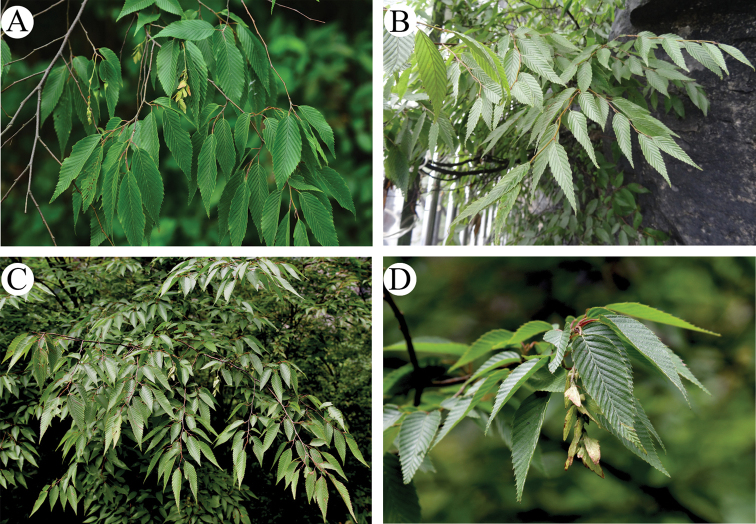
*Carpinus polyneura***A, B** population from Tianmenshan, Zhangjiajie City (type locality of *C. dayongina*) (**A** photographed by H. Zhou **B** photographed by W. Q. Qin) **C, D** population from Wushan, Chongqing City (near the type locality of *C. polyneura*) (**C, D** photographed by H. L. Zhou).

**Figure 3.**
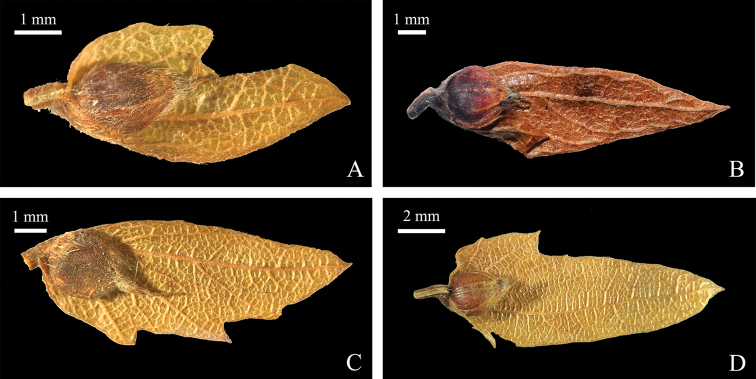
Nutlet trichomes of *C. polyneura***A** from isotype of *C. dayongina* (*K. W. Liu 33359*, PE01843387) **B** from paratype of *C. dayongina* (*Y. T. Xiao 40700*, CSFI017539) **C** from *T. L. Dai 104469*, PE00818275 **D** from *W. P. Fang 7546*, PE00818253. **A, C, D** photographed by Q. Q. He **B** photographed by X. Li.

Re-collections of material from the type localities and further field investigation showed that the leaf shapes are quite variable in the same area, from ovate-lanceolate to lanceolate, then narrow-lanceolate (Fig. 2). After a thorough examination of more specimens, we found that, not only leaf blade shape, but also infructescence length and bract size of *C. dayongina*, are all within the limits of *C. polyneura* (Table 5). After review of the type specimens, we found the leaf margins of *C. polyneura* and *C. dayongina* have both simply and doubly setiform serration (Table 5). In addition, by carefully searching for comprehensive and extensive groups of specimens, performing field investigations and measuring morphological characteristics, we found that the indumentum of their nutlets is also variable, from being villous at the apex and glabrous, sparsely villous or pubescent in the remaining part (Fig. 3, Table 5). Therefore, this character can also not be regarded as a useful character to differentiate these two taxa.

**Table 5.** Measurements of morphological characteristics of *Carpinus polyneura* and *C. dayongina*. A dash (–) indicates missing data.

Species	Specimen	Leaf length × width (cm)	Leaf size ratio	Leaf margin	Infructescence length (cm)	Bract size (cm)	Nutlet indumentum
*C. polyneura*	T. L. Dai 104469, PE00818275	5–5.9 × 1.7–1.8	2.9–3.3	Simply setiform serrate	2.6–2.9	1 × 0.35	Villous at apex
	H. F. Zhou 26421, KUN0590808	5.1–5.9 × 1.9–2.3	2.5–2.8	Simply setiform serrate	3.2–5.6	1.3 × 0.5	Villous at apex
	H. F. Zhou 26317, KUN0590809	5.5–6.2 × 1.7–1.9	3–3.4	Simply setiform serrate at apex, doubly setiform serrate in the rest	1.8–3.2	1.2 × 0.45	Villous at apex
	Z. R. Zhang 25054, KUN0590804	5.8–6.5 × 2.1–2.5	2.3–3.1	Simply setiform serrate	4.4–4.9	1.5 × 0.6	Villous at apex
	Y. Liu 668, KUN0590794	3.9–4.1 × 1.2–1.3	3–3.3	Simply setiform serrate	2.1	1 × 0.3	Pubescent
	P. C. Cai 20297, CSFI017457	5.3–6 × 1.8–2.1	2.8–3	Doubly setiform serrate	2.6–3.9	1.2 × 0.4	Pubescent
	C. L. Long 87290, CSFI017461	5.4–6.1 × 1.6–2.2	2.5–3.4	Doubly setiform serrate	3.9–4.3	1.2 × 0.5	Villous at apex
	J. R. Zheng 80108, CSFI017456	6.7–7.1 × 2.3–2.5	2.8–3	Doubly setiform serrate	–	–	–
	P. C. Cai 20442, CSFI017455	5.1–5.7 × 1.8–1.9	2.7–3	Simply setiform serrate at apex, doubly setiform serrate in the rest	2.2–2.7	1.2 × 0.5	Villous at apex
	P. Y. Li 8277, KUN0590814	5.9–7.3 × 2.2–2.7	2.4–3	Simply setiform serrate	4.4–4.8	1.4 × 0.6	Villous at apex
	T. P. Soong 39145, KUN0590798	5.5–6.5 × 2.4–2.7	2.2–2.7	Doubly setiform serrate	2–3.6	1.2 × 0.4	Villous at apex
	G. H. Yang 57149, KUN0590811	6–7.4 × 2.1–2.5	2.8–3	Simply setiform serrate, sometimes doubly setiform serrate	4.1–5.5	1.4 × 0.5	Pubescent
	W. P. Fang 7546, PE00818253	5–6.2 × 1.8–2.5	2.5–2.8	Simply setiform serrate	2.8	1.2 × 0.5	Villous at apex
	W. P. Fang et al. 31110, IBK00079496	6.7–7.5 × 1.9–2.1	3.2–3.7	Simply setiform serrate	3.1	1 × 0.3	Pubescent
	W. P. Fang et al. 32888, IBSC0368122	5.4–6.6 × 2.5–3	2–2.3	Doubly setiform serrate	3.2	1.5 × 0.5	Villous at apex
	G. H. Yang 54569, IBSC0368131	6–7.2 × 2–2.5	2.4–3.3	Doubly setiform serrate	3.9	1.4 × 0.5	Villous at apex
	S. G. Wu 394, KUN0590803	4.8–5.6 × 1.5–1.8	2.7–3.2	Simply setiform serrate	2.2–3.5	1.1 × 0.4	Villous at apex
	G. R. Chen 2383, KUN0590815	5.1–5.8 × 1.7–1.9	2.8–3	Doubly setiform serrate	2.8–4.1	1.1 × 0.4	Pubescent
	Anonymous 2759, KUN0590800	5.7–6.4 × 1.8–2.1	2.9–3.5	Doubly setiform serrate	3.1	1.3 × 0.4	Pubescent
	S. Y. Hu 1906, KUN0590797	6–6.6 × 2–2.2	2.7–3	Simply setiform serrate, sometimes doubly setiform serrate	5–6.5	1.5 × 0.6	Villous at apex
*C. dayongina*	Y. T. Xiao 40700, CSFI017539 (paratype of *C. dayongina*)	5.1–5.7 × 1.2–1.5	3.4–3.8	Simply setiform serrate	3.5–4.2	1.1 × 0.35	Villous at apex
	Y. T. Xiao 40700, CSFI017465 (paratype of *C. dayongina*)	5.7–6.5 × 2.3–2.8	2.2–2.7	Doubly setiform serrate	3.6–3.8	1 × 0.4	Villous at apex
	K.W. Liu 33359, CSFI050241 (holotype of *C. dayongina*)	4.3–4.7 × 1.4–1.6	2.9–3.6	Simply setiform serrate	2.5	0.7 × 0.4	Pubescent
	K. W. Liu 33359, PE01843387 (isotype of *C. dayongina*)	4.4–5 × 1.3–1.7	2.8–3.5	Simply setiform serrate	2.1	0.7 × 0.4	Pubescent
	Q. P. Zhang 2020072801, NF	5–5.6 × 1.6–1.8	2.7–3.3	Simply setiform serrate	–	–	–
	Q. P. Zhang 2020072802, NF	4.5–4.9 × 1.4–1.8	2.7–3.7	Simply setiform serrate, sometimes doubly setiform serrate	2	0.9 × 0.3	Villous at apex

**Figure 4.**
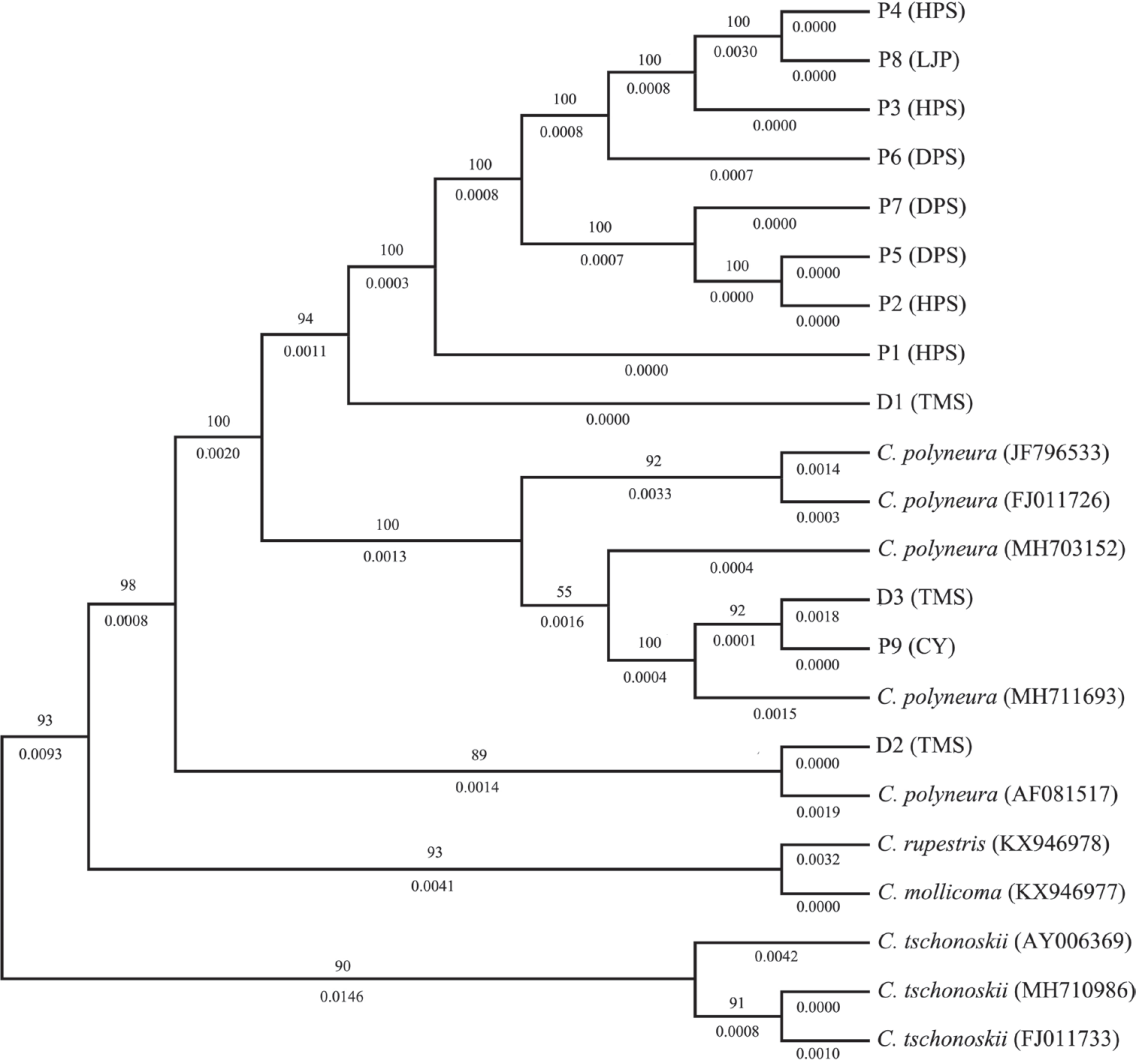
The neighbor-joining (NJ) tree, based on ITS sequence data. *Carpinus mollicoma*, *C. rupestris* and *C. tschonoskii* as related species; **P1–P4** are samples of *C. polyneura* from Hupingshan (HPS), Shimen, Hunan, China; **P5–P7** are samples of *C. polyneura* from Dapanshan (DPS), Panan, Zhejiang, China; **P8** is a sample of *C. polyneura* from Liujiaping (LJP), Wufeng, Hubei, China; **P9** is a sample of *C. polyneura* from Caoyuan (CY), Longli, Guizhou, China; **D1–D3** are samples of *C. dayongina* from Tianmenshan (TMS), Zhangjiajie, Hunan, China. The numbers above the branch are bootstrap values (%), and the numbers below the branch are branch length.

Phenetic comparison of ITS sequences showed that the samples of *C. dayongina* from Tianmenshan population and those of *C. polyneura* from other regions are mixed with each other (Fig. 4).

We therefore conclude that *C. dayongina* and *C. polyneura* are conspecific. According to ICN (McNeill et al. 2018), the earlier published *C. polyneura* has priority over *C. dayongina* and thus, *C. dayongina* is reduced to a synonym of *C. polyneura* herein.

### Taxonomic treatment

#### Carpinus polyneura

Taxon classificationPlantaeFagalesBetulaceae

Franch., J. Bot. 13: 202. 1899.

9DCC1826-F4A9-5CE0-8710-139CA5EEFD5B

≡ Carpinus turczaninovii var. polyneura (Franch.) H. J. P. Winkl., Das Pﬂanzenreich IV 61 (Heft 19): 38 (Winkler 1904). – Type: China, Su-tchuen [Sichuan], in District de Tchen kéou tin [Chengkou County], P. G. Farges s. n. (lectotype: P06811144!, designated by Holstein and Weigend 2017; isolectotypes: P06811145!, P06811146!) (Fig. 1A).
= Carpinus dayongina K. W. Liu & Q. Z. Lin, Bull. Bot. Res., Harbin 6(2): 143. 1986. syn. nov. – Type: China, Hunan, Dayong [now Zhangjiajie], Tianmenshan, 1100 m a.s.l., 28 July 1985, *K. W. Liu* [*Liu Ke Wang*] *33359* (holotype: CSFI050241!; isotype: PE01843387!) (Fig. 1B). – Additional original material: ibid., 25 August 1984, *Y. T. Xiao* [*Xiao Yu Tan*] *40700* (paratypes: CSFI017465!, CSFI017535!, CSFI017536!, CSFI017538!, CSFI017539!, CSFI017542!) (Figs 1C and D).

##### Description.

A deciduous tree, up to 15 m tall. Bark grey. Branchlets slender, dark purplish, covered with white roundish lenticels, densely villous and soon glabrous. Petiole 5–11 mm, 0.5–0.8 mm in diameter; leaf blade ovate-lanceolate to narrow-lanceolate, sometimes oblong or ovate, 2.5–8 × 1–3 cm, base broadly sub-rounded or slightly cordate, margin doubly setiform serrate, sometimes simply setiform serrate at the apex or simply setiform serrate, upper surface sparsely villous, densely villous along the veins, glabrescent, lower surface densely villous along the veins, sometimes bearded in axils of lateral veins, lateral veins 15–20 on each side of the mid-vein. Infructescence 1.4–6.5 cm long, pendent, peduncle slender, densely villous with white roundish lenticels; bracts semi-ovate-lanceolate, 5–15 × 3–6 mm, outer margin dentate, without basal lobe, inner margin entire with small, inflexed basal auricle. Nutlets broadly ovoid, 2–4 × 1.5–3 mm, villous at apex, glabrous or sparsely villous or pubescent on the remaining part, ribbed.

##### Distribution and habitat.

**China**: Chongqing, Gansu, Shaanxi, Sichuan, Guizhou, Hubei, Hunan, Jiangxi and Zhejiang. This species grows in subtropical broad-leaved forests or thickets at altitudes of 400–1900 m.

##### Additional specimens examined.

***Carpinus polyneura*:** Chongqing, Chengkou, 5 September 1958, *T. L. Dai 104405* (IBSC, NAS, PE, SZ); Chongqing, Chengkou, 6 September 1956, *T. L. Dai 104469* (IBSC, NAS, PE, SZ); Chongqing, Chengkou, 14 September 1958, *T. L. Dai 104783* (IBSC, NAS, PE, SZ); Chongqing, Chengkou, 20 September 1958, *T. L. Dai 104961* (IBK, NAS, PE); Chongqing, Fengjie, 1 June 1958, *Z. R. Zhang 25054* (HIB, IBSC, IFP, KUN, NAS, PE); Chongqing, Fengjie, 16 June 1958, *H. F. Zhou 26317* (HIB, IBSC, IFP, KUN, NAS, PE); Chongqing, Fengjie, 25 June 1958, *H. F. Zhou 26421* (HIB, IBSC, IFP, KUN, PE); Chongqing, Nanchuan, 8 June 1957, *J. H. Xiong & Z. L. Zhou 91297* (HIB, IBSC); Chongqing, Nanchuan, 29 June 1957, *J. H. Xiong & Z. L. Zhou 91753* (HIB); Chongqing, Wushan, 15 October 1958, *G. H. Yang 59826* (IBSC, PE, SHM); Chongqing, Wushan, 20 August 1964, *H. F. Zhou & H. Y. Li 110088* (IBSC, PE, SZ); Chongqing, Wuxi, 8 August 1958, *M. Y. Fang 23898* (HIB, IBSC, IFP, KUN, PE, SZ); Chongqing, Wuxi, 30 June 1958, *G. H. Yang 58658* (IBK, IBSC, PE, SHM); Gansu Province, Chengxian, 31 August 1958, *Z. P. Wei 2245* (HHBG, HNWP, SZ, WUK); Guizhou Province, Tungtze, 27 May 1930, *Y. Tsiang 5177* (IBSC, NAS, PE); Hubei Province, Badong, 31 October 1958, *S. X. Fu 1228* (HIB); Hubei Province, Hefeng, 27 August 1958, *H. J. Li 5874* (HIB, IBSC, KUN, PE, SZ); Hubei Province, Shennongjia, 29 August 1976, *Hubei Shennongjia Botanical Expedition 32766* (HIB, PE); Hubei Province, Shennongjia, 10 August 1976, *Hubei Shennongjia Botanical Expedition 31451* (HIB, PE); Hubei Province, Shennongjia, 19 June 1976, *Hubei Shennongjia Botanical Expedition 30417* (HIB, PE); Hubei Province, Shennongjia, 12 August 1976, *Hubei Shennongjia Botanical Expedition 11239* (HIB, PE); Hubei Province, Shennongjia, 26 August 1976, *Hubei Shennongjia Botanical Expedition 22775* (HIB); Hubei Province, Shennongjia, 3 June 1987, *Y. Liu 00668* (HIB, NAS, PE); Hubei Province, Shennongjia, August – September 1959, *Z. E. Zhao 113* (HIB); Hubei Province, Xingshan, 3 June 1957, *Y. Liu 668* (KUN, NAS, PE); Hunan Province, Anhua, 2 October 1978, *Z. H. Shen 1669* (CSFI); Hunan Province, Baojing, 8 August 1991, *X. L. Yu 91547* (CSFI); Hunan Province, Chengbu, 14 July 1981, *T. R. Cao 032* (CSFI); Hunan Province, Chengbu, August 1981, *Q. Z. Lin 11152* (CSFI); Hunan Province, Cili, 1 September 1984, *Xiangxi Expedition 0066* (PE); Hunan Province, Longshan, 10 August 1957, *B. M. Yang 2041* (IBSC, PE); Hunan Province, Luxi, 12 April 1982, *K. W. Liu 30079* (CSFI); Hunan Province, *Qianyang Expedition 122207* (IBK); Hunan Province, Sangzhi, 24 August 1988, *Beijing Expedition 3922* (PE); Hunan Province, Sangzhi, October 1976, *Sangzhi Expedition 960* (CSFI); Hunan Province, Shimen, 30 June 1979, *P. C. Cai 20297* (CSFI); Hunan Province, Shimen, 24 May 1987, *C. L. Long 87290* (CSFI); Hunan Province, Shimen, 4 May 1980, *J. R. Zheng 80108* (CSFI); Hunan Province, Yongshun, 31 May 1988, *Beijing Expedition 00571* (PE); Hunan Province, Yongshun, 5 June 1988, *Beijing Expedition 01123* (PE); Hunan Province, Yongshun, 22 August 1991, *X. L. Yu 91751* (CSFI); Hunan Province, Yongshun, 27 August 1991, *X. L. Yu 91854* (CSFI); Hunan Province, Yuanling, 23 June 1988, *Zhang et al. 512* (PE); Hunan Province, Yuanling, 23 June 1988, *G. C. Zhang et al. 510* (PE); Hunan Province, Yuanling, 15 June 1988, *Zhang et al. 380* (PE); Hunan Province, Zhangjiajie, 18 August 1979, *P. C. Cai 20442* (CSFI); Jiangxi Province, *anonymous 0470* (SHM); Jiangxi Province, Quannan, 27 June 1958, *anonymous 01184* (PE); Jiangxi Province, Shangrao, 21 June 1982, *anonymous 518* (LBG); Jiangxi Province, Xiushui, 4 September 1963, *S. K. Lai 03449* (LBG, SHM); Jiangxi Province, Zixi, 11 October 1985, *S. K. Lai & D. F. Huang 319* (LBG); Jiangxi Province, Zixi, 7 November 1957, *M. J. Wang et al. 2561* (NAS); Shaanxi Province, Langao, 27 July 1959, *P. Y. Li 8277* (KUN); Shaanxi Province, Zhenping, 13 May 1989, *G. Y. Xu 4899* (WUK); Shaanxi Province, Zhenping, 20 July 1991, *J. S. Ying et al. 217* (WUK); Sichuan Province, without date, *anonymous 20574* (IBK); Sichuan Province, without date, *anonymous 6255* (IBK); Sichuan Province, without date, *E. H. Wilson 5191* (IBSC); Sichuan Province, without date, *S. Y. Hu 1906* (KUN); Sichuan Province, without date, *S. G. Wu 394* (KUN); Sichuan Province, 23 November 1935, *W. G. Hu 8842* (IBK); Sichuan Province, 23 November 1935, *W. G. Hu 8832* (IBK, SZ); Sichuan Province, Baoxing, 1954, *Z. P. Song 39145* (IBSC, KUN, PE, WUK); Sichuan Province, Baoxing, 9 May 1954, *Z. P. Song 38130* (IBSC, KUN, PE, SHM, SZ); Sichuan Province, Dujiangyan, 29 October 1956, *Q. Li 47102* (IBSC, PE, SZ); Sichuan Province, Ebian, 28 October 1938, *T. N. Liou 12651* (WUK); Sichuan Province, Emeishan, 18 May 1929, *W. P. Fang 14651* (IBSC); Sichuan Province, Emeishan, 6 May 1931, *W. P. Fang 18625* (IBSC, SZ); Sichuan Province, Emeishan, 15 June 1952, *W. P. Fang et al. 31110* (IBK, IBSC, NAS); Sichuan Province, Emeishan, 13 September 1952, *W. P. Fang et al. 32478* (IBK, PE); Sichuan Province, Emeishan, 26 September 1952, *W. P. Fang et al. 32888* (IBK, IBSC, NAS, SHM); Sichuan Province, Emeishan, 6 September 1957, *G. H. Yang 57149* (HIB, IBSC, KUN, NAS, PE); Sichuan Province, Emeishan, without date, *Sichuan University Biology Department Expedition 54569* (HIB, IBSC, KUN, NAS, PE, SZ); Sichuan Province, Emeishan, without date, *Sichuan University Biology Department Expedition 52273* (HIB); Sichuan Province, Emeishan, without date, *Sichuan University Biology Department Expedition 51243* (HIB); Sichuan Province, Emeishan, 25 April 1940, *S. L. Sun 1711* (KUN, PE); Sichuan Province, Emeishan, 1 May 1940, *S. L. Sun 1800* (KUN); Sichuan Province, Emeishan, 17 May 1940, *S. L. Sun 1993* (KUN); Sichuan Province, Emeishan, 21 May 1940, *S. L. Sun 2127* (KUN, PE); Sichuan Province, Emeishan, 6 June 1931, *F. T. Wang 23224* (IBSC, PE, WUK); Sichuan Province, Emeishan, without date, *J. H. Xiong et al. 31690* (IBSC); Sichuan Province, Emeishan, sin.dat. *J. H. Xiong et al. 31790* (IBK); Sichuan Province, Emeishan, 10 May 1957, *G. H. Yang 54569* (IBSC, KUN, NAS, PE, SZ); Sichuan Province, Emeishan, 6 September 1957, *G. H. Yang 57149* (HIB, IBSC, KUN, NAS, PE, SZ); Zhejiang Province, Jiande, 4 July 1986, *C. R. Wu L8413260* (IBSC); Zhejiang Province, Tiantai, 1958, *G. R. Chen 2383* (KUN, PE); Zhejiang Province, Zhuji, 21 June 1984, *Y. J. Gao et al. 823-068* (IBSC).

## Supplementary Material

XML Treatment for Carpinus polyneura
